# Outbreak definition by change point analysis: a tool for public health decision?

**DOI:** 10.1186/s12911-016-0271-x

**Published:** 2016-03-12

**Authors:** Gaëtan Texier, Magnim Farouh, Liliane Pellegrin, Michael L. Jackson, Jean-Baptiste Meynard, Xavier Deparis, Hervé Chaudet

**Affiliations:** Centre Pasteur du Cameroun, BP 1274 Yaoundé, Cameroon; UMR 912/SESSTIM - INSERM/IRD/Aix-Marseille Université/Faculty of Medicine, 27, Bd Jean Moulin, 13385 Marseille, France; Institut Sous-régional de Statistique et d’Économie Appliquée, BP 294 Yaoundé, Cameroon; Center for Epidemiology and Public Health of the French Army (CESPA), Camp de Sainte Marthe, 13568 Marseille, France; Group Health Research Institute, 1730 Minor Ave, Suite 1600, Seattle, USA

**Keywords:** Outbreak identification, Change point analysis, Expert, Evaluation, Disease surveillance

## Abstract

**Background:**

Most studies of epidemic detection focus on their start and rarely on the whole signal or the end of the epidemic. In some cases, it may be necessary to retrospectively identify outbreak signals from surveillance data. Our study aims at evaluating the ability of change point analysis (CPA) methods to locate the whole disease outbreak signal. We will compare our approach with the results coming from experts’ signal inspections, considered as the gold standard method.

**Methods:**

We simulated 840 time series, each of which includes an epidemic-free baseline (7 options) and a type of epidemic (4 options). We tested the ability of 4 CPA methods (Max-likelihood, Kruskall-Wallis, Kernel, Bayesian) methods and expert inspection to identify the simulated outbreaks. We evaluated the performances using metrics including delay, accuracy, bias, sensitivity, specificity and Bayesian probability of correct classification (PCC).

**Results:**

A minimum of 15 h was required for experts for analyzing the 840 curves and a maximum of 25 min for a CPA algorithm. The Kernel algorithm was the most effective overall in terms of accuracy, bias and global decision (PCC = 0.904), compared to PCC of 0.848 for human expert review.

**Conclusions:**

For the aim of retrospectively identifying the start and end of a disease outbreak, in the absence of human resources available to do this work, we recommend using the Kernel change point model. And in case of experts’ availability, we also suggest to supplement the Human expertise with a CPA, especially when the signal noise difference is below 0.

**Electronic supplementary material:**

The online version of this article (doi:10.1186/s12911-016-0271-x) contains supplementary material, which is available to authorized users.

## Introduction

The US Centers for Disease Control and Prevention (CDC) define an epidemic as "the occurrence of more cases of disease than expected in a given area or among a specific group of people over a particular period of time"[1]. Because the course of many epidemics can be altered through early public health action, considerable research has been directed towards early detection of epidemics using public health surveillance data [2–4]. Nearly all of this work has focused on detecting the start of the epidemic, in order to initiate a timely response. However, identifying the full time course of an epidemic is useful for several reasons. First, identifying the end of the epidemic helps public health officials know the when response activities can cease. Second, defining the end of an epidemic in disease surveillance helps determine whether new cases are part of a known or a new outbreak. The notion of end of epidemic underlies also a large literature on disease elimination or eradication [5–8] and (re-) emerging pathogens. From an economical point of view, declaring the end of an epidemic impacts trade and tourism. INSEE (France's National Institute of Statistics and Economic Studies) evaluated the consequences on tourism of the Chikungunya outbreak that occurred in La Reunion Island in 2006. The result was a decrease of 130 000 visitors compared with 2005, a general increase of 8 % of unemployment in tourism activities, growing to 25 % for the only hostel trade. The total cost of the public aid for 2006 was evaluated to 20 millions euros for this activity [9]. Finally, detecting the full extent of past outbreaks in surveillance data can improve future outbreak identification. When undetected prior outbreaks are included in surveillance baseline data, the surveillance baselines are biased upward, and future outbreaks will be more difficult to detect [10, 11].

Our study aims at evaluating the ability of change point analysis (CPA) methods to identify the beginning and ending dates of a disease outbreak from weekly counts. We will compare our approach with the results coming from experts’ signal inspections, considered by many as the gold standard method.

## Background

### Human signal inspection

Historically, identifying a whole outbreak signal in surveillance data has relied more on human judgment, for example through review by a committee of experts [12], than on signal processing as for the prospective detection of outbreak starts. This visual inspection of the time series is still considered by many authors as the gold standard approach. But the difficulty to find available experts for this task, the evidence of variability in opinion among the experts, and the poor knowledge about the factors influencing this variability (Watkins 2006 [13]) lead us to say with Buckeridge [14] that this method can be considered as a “time-consuming procedure whose reliability is unknown”. All these reasons raise the interest for a statistical approach.

### Change point analysis

#### Epidemics as state changes in surveillance series

Following several authors [15–19], we consider that the weekly count of an infectious disease is a time series resulting from a model combining two endemic and epidemic components. If no outbreak occurs, the endemic component is alone. A complete time series can be then seen as a succession of model regimes corresponding to the alternation of no outbreak (endemic) and outbreak (epidemic) state. The identification of a regime change associated with its time in the series is known as the “change point problem”. Indeed, considering a time series {x_1_, x_2_,…, x_n_} measured with an index of time τ ∈ {1, 2,…,n}, a change point is a time index where a structure change occurs in data.

As all previous authors working on use of CPA in the context of surveillance data of infectious disease counts [15–19], we will assume that the endemic or epidemic component of the process is driven by parameter (as mean and/or variance incidence) which were considered as piecewise constant. The epidemic change point problem is a special multiple change point problem developed in Chen 2011 [20]. Identifying an epidemic regime requires to identify the endemic states that come before and after this epidemic segment. In this context, we need to identify pre-epidemic period (endemic state), epidemic period (epidemic state) and post-epidemic period (endemic state) building a free state model.

#### Outbreak change point problem formulation

A change point model is a model assuming the existence of at least one change point (at the time index τ when the signal has changed) and partitioning the data into disjoint segments (with parameters similar within each segment and different without). This analysis is recursively repeated for each segment, allowing the detection of multiple changes.

Usually the state change occurs at an unknown time index τ. It is the reason why the problem is formulated using a change point detection (detection formulation: “How many changes during the time series?”) and a change point estimation (locations formulation: “When do they occur within the time series?”)

Let {x_1_, x_2_,…, x_n_} a time series of independent random variables and θ_i,_ i = 1,…, n the corresponding structure parameters. The change-point analysis of the time series consists in the following two steps:

1. Decide between

*H*_0_ : *θ*_1_ = ⋯ = *θ*_*k*_ = ⋯ = *θ*_*τ*_ = ⋯ = *θ*_*n*_ No change point

and

*H*_1_ : *θ*_1_ = ⋯ = *θ*_*k*_ = *α* ≠ *θ*_*k* + 1_ = ⋯ = *θ*_*τ*_ = *β* ≠ *θ*_*τ* + 1_ = ⋯ = *θ*_*n*_ = *γ* Change points

where 1 < k < τ < n, and α, β, γ are unknown, k and τ representing the start and end dates of the outbreak.

A change point is detected when H_0_ is rejected.

The next step of change point estimation is carried out only when the null hypothesis of no change point is rejected. The number of change points and their respective unknown positions has to be estimated.

2) Estimate k and τ from the sample {x_1_, x_2_,…, x_n_}, if H_1_ is true.

Depending on the underlying model used to solve the change point problem, we may distinguish parametric (based on Maximum Likelihood) non-parametric (Kernel, Kruskall-Wallis) and Bayesian change point model.

## Methods

All the experiments were performed using R 3.1.1 [21].

To evaluate precisely the ability of change point analysis methods to identify a whole outbreak time curve, we need to build a gold standard by controlling perfectly when the outbreak starts in time series and when it ends. In the absence of a consensual gold standard defined in real world, only simulated data allow this evaluation.

### Outbreak generation

For the realism of the generated outbreak curve, as proposed by Jackson [22], we used an Inverse Transform Sampling Method algorithm (ITSM) to simulate the signal from a real outbreak of Norovirus already published [23]. According to these outbreaks, we generated 100 curves for each outbreak magnitude (10, 30, 50, 100 cases) while conserving the original number of day (*n* = 12). Due to the use of a probabilistic process to generate this curve, the resulting duration of the simulated outbreak can be shorter than the original one, in particular for outbreak with small number of cases (*n* = 10).

### Baseline generation

Seven levels of baselines were generated, corresponding to the expected daily incidences of 0, 1, 3, 5, 10, 20, 30, with 0, 0.25, 2.25, 5, 15, 50, 100 as associated variances. The baseline durations were 72 days for the Norovirus. For each incidence level we randomly generated 200 baselines according to a Gaussian law.

### Evaluation data sets

With an objective of result reproducibility, we choose to build, for each combination of outbreak magnitude and baseline level of daily incidence, a data set of 30 times series. Each time series was created by adding a randomly selected outbreak among the 100 to a randomly selected baseline among the 200. For fully controlling the beginning and ending dates of the outbreak within the time series, we systematically added the outbreak after the first 30 days of baseline and kept 30 days after the outbreak end. Finally, 840 time series (4 level of curves x 7 level of baseline x 30 replicates) in 28 data sets were produced for evaluating the algorithms.

The building process was slightly different concerning the human experts. For avoiding a process of learning, we have chosen to randomly place the epidemic period in each series, with the constraint of keeping at least 10 days of baseline before the outbreak beginning and 10 days of baseline after the outbreak ending. Taking in account the workload, we built 28 data sets of 2 time series only. However, we have randomly reordered the 56 resulting time series presented to each expert for controlling a possible ordering effect.

### Expert evaluation

We enlisted 15 experts who have at least 1 year’s experience in daily disease surveillance. To allow comparison with the algorithms, we gave the experts the information that one and only one outbreak was present in each time series. Each expert independently evaluated 56 time series. The time series were presented in both graphical and numerical format (see Figure S1 in Additional file 1). Experts were asked to visually identify the beginning and ending dates of the simulated outbreaks without the aid of calculators, spreadsheets, or other tools. Results from all the experts were pooled to treat the expert review as a single unique “algorithm” analyzing a total of 840 times series.

### CPA models

#### Maximum likelihood CPA model

As proposed by Chen 2011 [20], an efficient strategy to identify change-points is to select the partition of the time series sample {x_1_, x_2_,…, x_n_} that yields a maximum of heterogeneity between segments. This method tries to cut the time series {x_1_, x_2_,…, x_n_} by maximizing the likelihood in 3 continuous states {x_1_, x_2_,…, x_τ1_}; {x_τ1+1_, x_τ1+2_,…, x_τ2_} and {x_τ2+1_, x_τ2+2_,…, x_n_} described previously. If this 3 states are confirmed, and if we consider that the time series is organized into 2 endemic periods encircling a epidemic period, τ_1_ and τ_2_ correspond to interesting change points: the start and end dates of the outbreak. Because x_τ_ are counting data (number of cases), we can hypothesize that x_τ_ follows a Poisson distribution. Even if infectious disease data are often overdispersed, this distribution hypothesis may be kept, as it usually done in disease surveillance [19], and to ensure evaluation fairness between algorithms. The detailed method is presented in Additional file 1.

#### Kernel CPA model

This CPA is detailed in Harchaoui et al. [24].

Let {x_1_, x_2_,…, x_n_} a time series of *independent* random variables. We can define a Kernel function K as:

$$ {y}_i=K\left({x}_i\right)\kern0.5em \forall\ i\ \in\ \left\{1,2,\dots, n\right\} $$

In a second time we can define a Kernel Fisher discriminant ratio (KFDR), which measures the heterogeneity between the successive segments S_1_, S_2_, S_3_

- S_1_ = {x_1_, x_2_,…, x_i_} with i observations,: pre-epidemic
- S_2_ = {x_τ1+1_, x_τ1+2_,…, x_j_} with (j-i) observations,: epidemic
- S_3_ = {x_τ2+1_, x_τ2+2_,…, x_n_} with (n-j) observations.: post-epidemic

A kernel function is required to calculate this KFDR. In literature, many functions exist (Gaussian, Laplace…) for k, but we choose for this work a simple linear kernel function: *k(x,y) = xy* that amounts calculating a Fisher test statistic to compare the heterogeneity between S1, S2, S3. The detailed method is presented in Additional file 1.

#### Kruskal-Wallis model

Tests against the epidemic alternative H_1_: ”a change occurs in the baseline“ with a non-parametric technique are proposed in Yao (1993) [25] and Emad-Eldin (1996) [16]. Adapted to our hypothesis, let L_1_ the law of the random variable X on the first segment (pre-epidemic), L2 the law on the second segment (epidemic) and L_3_ the law on the last segment (post-epidemic). The Kruskal-Wallis test is used to check the following hypotheses:

- H_0_: L_1_ = L_2_ = L_3_.
- H_1_: Laws L_1_, L_2_, and L_3_ are not identical.

The detailed method is presented in Additional file 1.

#### Bayesian model

The Bayesian model used here is the one introduced in 1998 by Siddhartha Chib [26]. Chib proposes to introduce a latent variable (S_t_) that takes discrete values from 1 to the total number of hidden regimes (*m*) in the series. Each discrete value indicates the kind of data-generating regime at each time unit (τ). This approach allows reproducing the epidemic and non epidemic latent regimes that generate the disease surveillance time series X_n =_ {x_1_,x_2_,…,x_n_}, with n observations. This Bayesian approach ensures some flexibility by using a limited number of dependent variables, while keeping the capacity of managing multiple change points [27].

Let x_t_ the number of events at time t and *m* a hidden state (or regime) at t. The x_t_ distribution according X_t-1_ = {x_1_,x_2_,…,x_t-1_} depends on the transition parameters (transition kernel) ξ_t_, which values {θ_1_,θ_2_,…,θ_m_} change at unknown dates {τ_1_, τ_2_,…,τ_m-1_}. In Chib’s model, the transition of hidden states is constrained to move forward by a non-ergodic Markov chain that makes the regime changes irreversible. Discrete variable S_t_ is modeled by a Markov chain process with probability matrix *P*, without possibility to return back to a previous state. The detailed method is presented in Additional file 1.

Data analysis was done with the MCMCpoissonChange function provided by MCMCpack [28].

### Evaluation metrics and signal noise difference

We evaluated the algorithms using several metrics.

The beginning detection delay (d_1_), measured in days, is equal to the absolute value of the difference between the start date according the algorithm and the real start date.

The ending detection delay (d_2_), measured in days, is equal to the absolute value of the difference between the end date according the algorithm and the real end date.

As a general measure of accuracy we used an overall detection error, measured in days, defined as the sum of all the delays considering d_1_ and d_2_ with the same importance:

$$ \mathrm{Error} = \left|{\mathrm{d}}_1\right|+\left|{\mathrm{d}}_2\right| $$

The specificity (Sp) expresses the capability to consider a day as non-epidemic while it is really not epidemic.

The sensitivity (Se) expresses the capability to consider a day as an outbreak day while it is really epidemic.

For the last metric we selected the Bayesian probability of correct classification. Our detection problem consists in deciding which is the true binary state of a day in the time series (baseline or outbreak), given the binary result of the algorithm for this day. If the 2 possible realizations for a day are noted H_0_ (non epidemic day) and H_1_ (epidemic day), their prior probabilities are P_0_ and P_1_. The Bayesian risk associated with this binary detection problem is then:

$$ \mathrm{Bayesian}\ \mathrm{Risk} = {\mathrm{C}}_{00}.{\mathrm{P}}_0.\mathrm{P}\left({\mathrm{H}}_0/{\mathrm{H}}_0\right) + {\mathrm{C}}_{01}.{\mathrm{P}}_1.\mathrm{P}\left({\mathrm{H}}_0/{\mathrm{H}}_1\right) + {\mathrm{C}}_{10}.{\mathrm{P}}_0.\mathrm{P}\left({\mathrm{H}}_1/{\mathrm{H}}_0\right) + {\mathrm{C}}_{11}.{\mathrm{P}}_1.\mathrm{P}\left({\mathrm{H}}_1/{\mathrm{H}}_1\right) $$

where C_.._ are the costs associated to each possibility and P(H_._/H_._) are the conditional probabilities for each realization.

If C_00_ = C_11_ = 1 and C_01_ = C_10_ = 0, the Bayesian risk is the binary Bayesian probability of correct classification (PCC), or the probability of exact decision.

To account for the influence of the outbreak and baseline sizes in the evaluation results we have used the difference between the signal (outbreak) and the noise (baseline), and not the signal to noise ratio as usual. This was required because of the existence of null baselines in the datasets. This signal to noise difference (SND) is defined as:

$$ \mathrm{S}\mathrm{N}\mathrm{D} = \mathrm{sum}\ \mathrm{of}\ \mathrm{outbreak}\ \mathrm{cases}\ \left(\mathrm{signal}\right)\ \hbox{--}\ \mathrm{sum}\ \mathrm{of}\ \mathrm{baseline}\ \mathrm{cases}\ \mathrm{during}\ \mathrm{the}\ \mathrm{outbreak}\ \mathrm{period}\ \left(\mathrm{noise}\right) $$

A positive SND corresponds to a higher number of cases in the outbreak than in the baseline during the outbreak period, and a negative one to the opposite.

## Results

### Accuracy Evaluation

#### Overall evaluations

Across all algorithms, the baseline and outbreak sizes affect accuracy and dispersion (Table 1). Larger outbreaks are associated with lower d_1_ and d_2_, while the inverse is true for larger mean baseline counts. Delay d_1_ (d_2_) goes from 13.4 to 2.4 days (11.7 to 2.8 days) when the outbreak size grows from 10 to 100 cases, and from 0.5 to 20.9 days (0.5 to 21.1 days) when the baseline level grows from 0 to 30. The precision of the dates increases with the outbreak size (16.5 to 1.1) and decreases when the baseline level grows (16.3 to 0.4).

**Table 1:** Algorithm accuracies according to the outbreak sizes and baseline levels

	Max- likelihood		K- Wallis		Kernel		Bayes		Expert	
	d_1_	d_2_	d_1_	d_2_	d_1_	d_2_	d_1_	d_2_	d_1_	d_2_
Outbreak sizes										
10	9.8 *(10.4)* ^*a*^	10.0 *(10.1)*	9.8 *(9.9)*	10.1 *(10.0)*	8.1 *(9.3)*	8.5 *(9.3)*	18.8 *(12.6)*	18.4 *(12.9)*	13.4 *(16.5)*	11.7 *(14.5)*
30	6.2 *(8.6)*	6.6 *(8.5)*	8.0 *(9.9)*	8.1 *(10.1)*	5.4 *(8.2)*	6.0 *(8.0)*	13.6 *(12.9)*	14.4 *(13.7)*	7.3 *(11.3)*	7.1*(11.2)*
50	4.0 *(6.2)*	4.3 *(5.9)*	4.8 *(7.2)*	4.9 *(7.2)*	3.5 *(4.9)*	3.9 *(4.7)*	9.9 *(11.9)*	10.3 *(12.4)*	5.4 *(9.3)*	4.5 *(8.1)*
100	2.4 *(1.2)*	2.8 *(1.2)*	3.2 *(4.9)*	3.1 *(4.8)*	2.6 *(1.1)*	3.2 *(1.0)*	5.3 *(8.4)*	5.3 *(8.3)*	3.9 *(7.5)*	3.2 *(4.5)*
Baseline levels										
0	1.5 *(0.9)*	1.6 *(1.0)*	1.1 *(0.5)*	1.1 *(0.4)*	1.9 *(1.1)*	2.5 *(1.4)*	1.5 *(0.9)*	1.5 *(1.0)*	0.5 *(1.1)*	0.5 *(1.2)*
1	1.7 *(1.0)*	2.1 *(1.2)*	1.8 *(2.0)*	1.6 *(1.8)*	1.9 *(1.2)*	2.4 *(1.3)*	5.7 *(9.9)*	5.2 *(8.4)*	2.5 *(6.4)*	1.6 *(1.6)*
3	3.9 *(6.6)*	4.3 *(6.7)*	4.6 *(7.5)*	4.6 *(7.4)*	3.1 *(5.1)*	3.7 *(5.3)*	9.7 *(12.6)*	10.0 *(13.0)*	4.3 *(8.0)*	4.6 *(9.0)*
5	4.5 *(6.8)*	4.9 *(6.4)*	6.0 *(7.7)*	6.1 *(8.0)*	3.5 *(5.2)*	4.0 *(5.0)*	11.9 *(12.7)*	12.2 *(12.5)*	7.7 *(12.4)*	6.8 *(10.9)*
10	6.6 *(8.6)*	6.9 *(8.2)*	*8.8 (9.9)*	9.0 *(9.6)*	5.5 *(7.7)*	5.9 *(7.5)*	15.8 *(12.0)*	17.0 *(12.9)*	9.8 *(12.5)*	8.8 *(11.6)*
20	10.0 *(9.9)*	10.3 *(9.6)*	10.7 *(10.1)*	10.2*(9.8)*	7.9 *(8.8)*	8.4 *(8.4)*	17.5 *(12.1)*	17.5 *(12.7)*	12.5 *(13.5)*	11.7 *(12.5)*
30	10.9 *(9.6)*	11.3 *(9.3)*	12.2 *(9.7)*	13.2*(10.3)*	10.3 *(9.6)*	10.9 *(9.5)*	20.9 *(11.1)*	21.1 *(12.2)*	15.4 *(16.3)*	12.5 *(13.7)*
Overall	5.6 *(7.9)*	5.9 *(7.7)*	6.5 *(8.6)*	6.5 *(8.8)*	4.9 *(7.0)*	5.4 *(6.9)*	11.9 *(12.6)*	12.1 *(12.9)*	7.5 *(12.2)*	6.6 *(10.8)*

^a^absolute mean (standard deviation).δ_1_ = Beginning date difference*/*δ_2_ *=* end date difference

The Kernel algorithm provides the more precise and unbiased global results according to d_1_ (σ =7.0, mean =4.9) and d_2_ (σ =6.9, mean = 5.4).

**Fig. 1:**
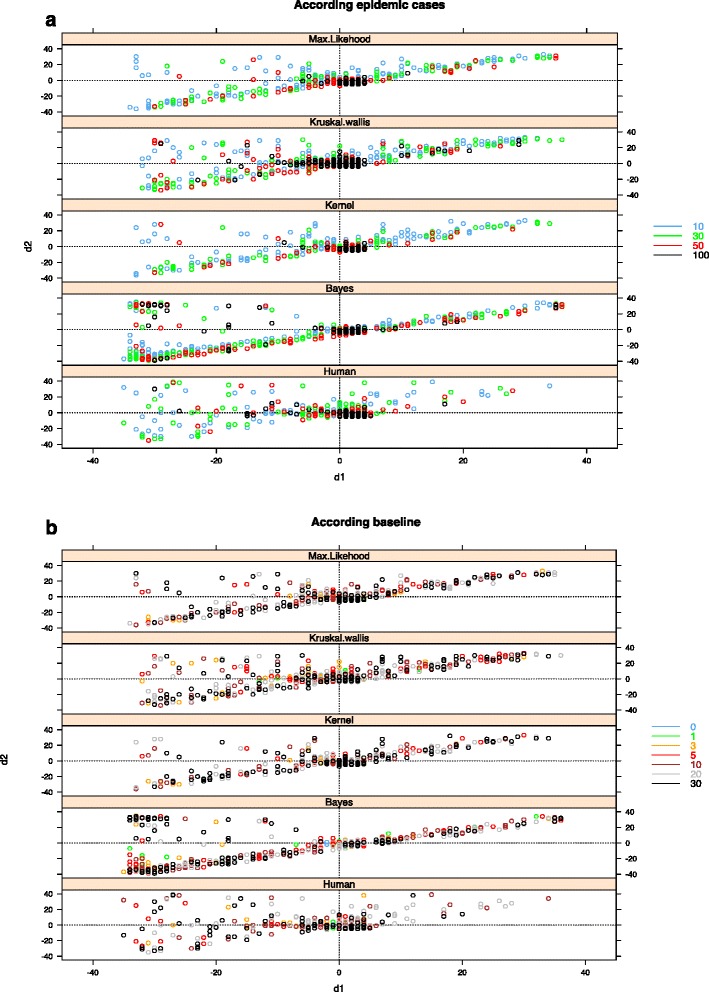
Algorithms (Maximum Likelihood, Kernel, Kruskall-Wallis, Bayesian, Expert) accuracy for 840 evaluations according to the outbreak size (**a**) and baseline level (**b**)

Figure 1 illustrates the influence of the baseline level and outbreak size on the combined d_1_ and d_2_ errors across the 840 time series. No point is below the first diagonal with a 4-days offset (the minimal outbreak duration) because this area corresponds to the impossible case of an outbreak beginning after its ending. The maximum likelihood and the kernel algorithms show their results mainly along this diagonal. This reveals that these algorithms try to find the shortest outbreak possible while exploring the time series. In contrast, the experts’ results are mainly grouped around the true values and do not show a specific alignment along the first diagonal.

The top left quadrant in each figure corresponds to the situations where all the real outbreak is included by excess within the detected outbreak, meaning that algorithm make an error not only for d_1_ but also for d_2_. It is most frequent for the Kruskal Wallis (26 % of the results) and Bayes (18 %) algorithms, and less frequent for the kernel (12,5 %) and the Max-Likelihood (16 %) algorithms, and least frequent for the experts (10 %).

**Fig. 2:**
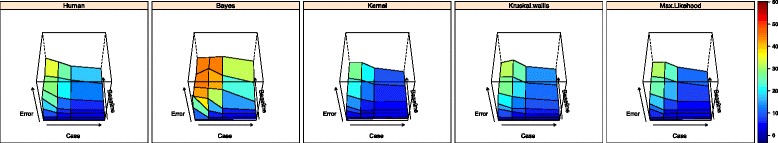
Algorithms accuracy evaluations (total error in days) according number of cases in the outbreak and level of baseline

The Kernel algorithm has the lower overall detection error (considering with the same importance d_1_ and d_2_ errors), whatever the outbreak size and the baseline level (Fig. 2).

#### Influence of baseline fluctuations

We focused on the CPA results with an error equal or greater than 2 days and then 4 days. Suspecting an effect of the random fluctuations of the baseline, we applied the algorithms on the only baselines and compared the results with the corresponding complete time series. The dates were the same in 34.8 % of the series with an error equal or greater than 2 days and 58.5 % of the series with an error equal or greater than 4 days.

#### Signal-noise influence

**Fig. 3:**
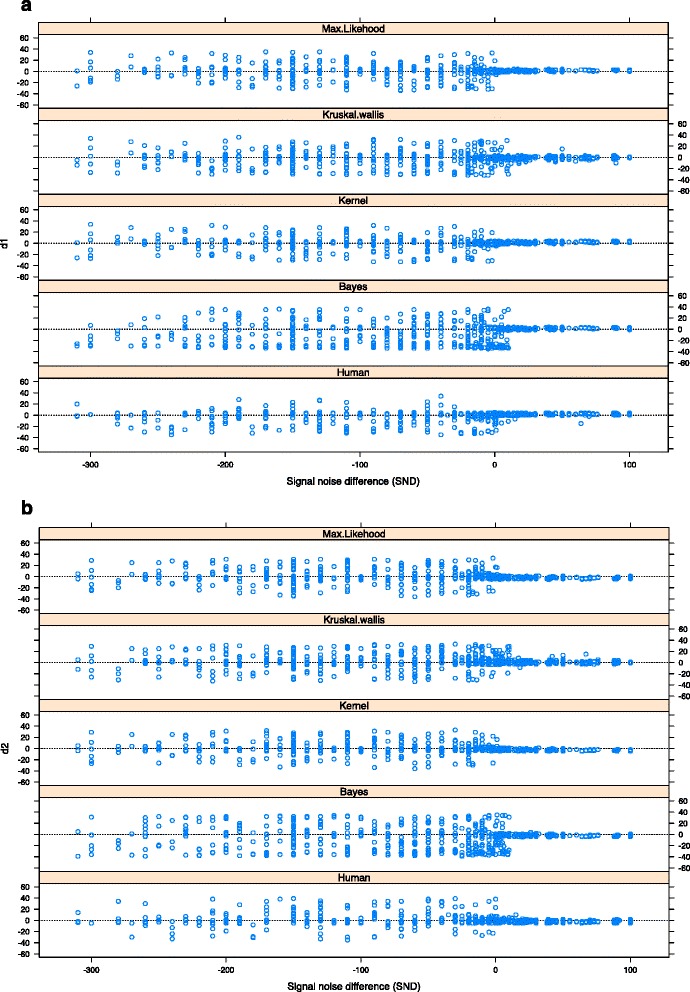
Impact of SND (signal noise difference) on algorithms (Maximum Likelihood, Kernel, Kruskall-Wallis, Bayesian, Human) accuracy measured by the difference with the real date (**a** d_1_ = Beginning date difference. **b** d_2_ *=* end date difference)

All algorithms show an important decrease in their capacity to identify d_1_, d_2_ when the SND goes under 0 (when the outbreak size is lower than the cumulated baseline during the outbreak period) (Fig. 3). The loss of accuracy starts before this threshold for the Bayes and Kruskall-Wallis algorithms and for the experts. There is a general tendency to delay the detection of the outbreak beginning and to anticipate the detection of its ending. Additional file 1: Table S1 supplements these results, showing that kernel and max-likelihood algorithms are the less biased and dispersed.

**Fig. 4:**
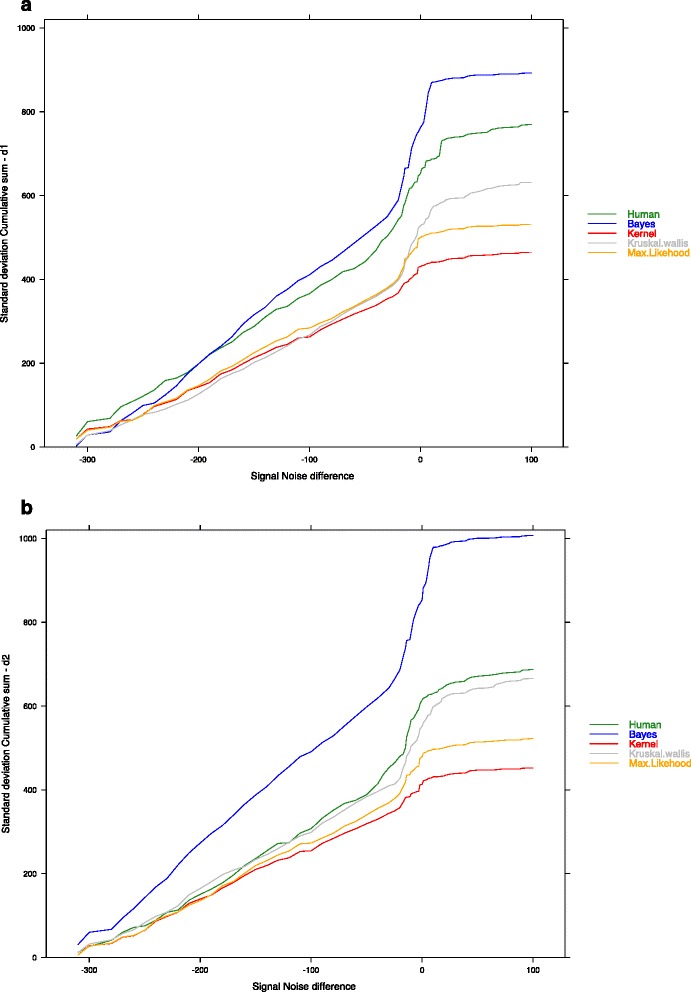
Impact of SND (signal noise difference) on algorithms (Maximum Likelihood, Kernel, Kruskall-Wallis, Bayesian, Human) error cumulated accuracy measured by cumulated standard deviation (**a** d_1_ = Beginning date difference. **b** d_2_ *=* end date difference)

The cumulative standard deviation (used as a proxy of the cumulative dispersion of the date detection) according to SND shows that the accuracy decreases with SND (Fig. 4), the slope being the accuracy loss rate. The curves clearly show 2 regimes with a slow increase of the dispersion when the number of cases in the outbreak is equal or higher than in the baseline during the outbreak period. It is possible to rank the algorithms and to define 3 groups: Kernel and Max-likehood, Human and Kruskall-Wallis, and Bayes. This decrease of accuracy is less important for the Kernel algorithm than for the other ones.

### Global detection performance

#### Sensitivity and specificity

The algorithm sensitivity and specificity are highly influenced by the outbreak size and the baseline level (Additional file 1: Figure S2), but the Kernel and Maximum likelihood algorithms have the best results in term of specificity (resp. 0.957 and 0.949) and the Kruskall-Wallis algorithm in term of sensitivity (0.675). Only the Bayes algorithm and some experts have sometime considered all the time series as the signal to detect (null specificity). This situation has been encountered with small outbreak sizes and high baseline levels. However, the CPA algorithms show global results characterized by high specificity associated with low sensitivity. In this sense these algorithms can be seen as conservative.

#### Probability of correct classification

**Fig. 5:**
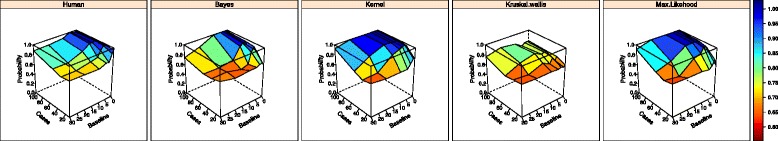
Probability of correct classification associated with each algorithm (Maximum Likelihood, Kernel, Kruskall-Wallis, Bayesian, Human) according the number of cases in the outbreak and the level of baseline

The probability of correct classification (PCC) can be considered as a probability for an algorithm to provide the good decision (according the real days status: endemic or epidemic) for all days in a time series. A PCC equal to 1 correspond to perfect status identification for the whole time series. Kernel perform globally better (with a PCC = 0.904) than Max-Likelihood (PCC = 0.903), Kruskall-Wallis (PCC = 0.884), Bayes (PCC = 0.862) and Human (PCC = 0.848). Detailed results are presented in Fig. 5. We noticed that Experts outperform algorithms for time series with low-level baseline, such as 0 (Experts PCC = 0.985, Kernel PCC = 0.94) or 1 (Experts PCC = 0.944, Kernel PCC = 0.94).

#### Time

On average, an expert could process 56 curves per hour (range, 36 to 105). An average of 15 h is then required to manually process all the 840 curves. In contrast, the slowest algorithm handles the whole set in 24 mn 37 s (Kruskall-Wallis), with a minimum of 3 mn 18 s for the Max-Likelihood algorithm.

## Discussion

Buckeridge [14] wrote that Experts can be considered as a “time-consuming procedure whose reliability is unknown” to identify epidemics. And if we agree with the fact that expert should be considered as a weakness for this purpose, the issue is to find an alternative and to evaluate its practicability.

Concerning the time-consuming aspect, a recent study [29] by Debin et al. assessed the capacity of experts to identify the starts and ends of influenza outbreaks with a web-based Delphi survey. The time required to mobilize the experts (*n* = 69 recruited among 288 eligible), to assess 34 times series, and to obtain a consensus was approximately 3 months (without including the time needed for recruiting the experts).

Although data-processing time is hardware configuration dependent we notice, only for comparison purpose, that expert need at least 15 h to assess 840 curves, in contrast with the maximum of 25 min and minimum of 3 mm 18 s of processing time for the CPA models. Taking in account the workload, the small number of skilled experts, and the probable volunteer fatigue as a result of solicitation increase, it is clear that considering expert review can be only an occasional solution for research purpose and is unusable in a routine context (surveillance and public health context).

Considering the reliability of the different methods (human and statistics), we tried to identify their limits. Our approach was to control the characteristics of the time series submitted to the different methods of outbreak identification by injecting controlled signals coming from real outbreaks within well-known baselines. Among the factors most influencing the evaluation results, we showed that the excess of case in the time series linked to the epidemics, represented by the SND, is the most pertinent factor and emphasizes the interaction between the outbreak size and the baseline level. Concerning accuracy, a drop out point, which is associated with an important accuracy decrease and dispersion increase and which is easy to materialize by the cumulative dispersion, can characterize each algorithm. Experts outperform the algorithms in term of PCC for low level of baseline but this ability quickly declines when SND decreases. Kernel and Maximum-Likelihood are the most accurate and less biased algorithms when the SND decreases under 0. All other algorithms loss their capacities as soon as SND reach 10. Kernel and Maximum-Likelihood can be considered, whatever the baseline level and outbreak size, as the best algorithms for providing a correct classification of the days in the time series according to their real status. For all algorithms, we observed a disposition to delay the detection of the first outbreak day and to anticipate the detection of the last outbreak day, probably in association with the slope of the outbreak curve.

Among the differences observed between Experts and algorithms, we noticed that when an Expert doesn’t find the outbreak signal he has a propensity to include the whole time series as the outbreak signal (increasing his sensitivity). Another individual variability observed in the Expert population, and increasing the result dispersion, is the way the instructions are understood and assimilated. In example, considering the instruction “End date for the outbreak is the last day always included in the epidemic signal but before a return to a normal situation” and the 0-level baseline, some Experts included in the outbreak signal the first baseline days (0 case) after the ending day, while in the same time considering outbreak cases occurring ahead of time as baseline noise and excluding them from the outbreak. As suggested by Wagner [4], this phenomenon may be explained by a possible mixing of detection and intervention objectives in the Experts’ cognitive processes. The need to have the same experimental condition for Experts and algorithms drove us to give the Experts the only time series without contextual information (as the agent or the target population) and to force them to give a result, even if they did not precisely identify the outbreaks. In reality, we believe the Experts are able to increase their performance in using information about the context and simple tools during a situational diagnosis (i.e. using the nature of the agent for inferring a plausible outbreak duration or size).

We also observed some limitations of both expert and CPA when the outbreak starts during a low level of the baseline fluctuation, making impossible the identification of the true beginning date.

Our study shows that nonparametric and parametric methods are more accurate and less biased than Bayesian CPA. Kass-Hout et al found the same result [30] and presumed it was because Bayesian CPA makes the assumption of a normal distribution of the time-series data, adopting non-informative priors on the model parameters. But in reason of the nature of disease surveillance data, the normality assumption may interfere with the estimation of the posterior distribution and the results obtained by the Bayesian CPA. Kernel algorithm has the most effective overall features in term of accuracy, bias and global decision concerning the outbreak day identification.

All this work has been done on detrended signals, without seasonality or autocorrelation. However, using a 3 states model, we integrate already the possibility of trend existence, as this allows the baseline to have different levels before and after the outbreak. Taylor [31] considers that only high levels of autocorrelation can influence the CPA results. In a same way, Zou wrote that if CPA remains useful in disease surveillance it is probably because most outbreaks are not strongly correlated in a limited time period. See Kass-Hout et al [30] for a larger discussion on influence of autocorrelation and trend on CPA model results in disease surveillance.

Despite the numerous advantages of the CPA algorithms, three main problems can be raised. Firstly, they are unable to detect unique aberrations in time series, as a single peak outbreak (not evaluated here), which are still easy to identify by an expert and usually best detected by usual time aberration detection methods. It's a reason why we recommend that CPA models should be compared to threshold-based methods in future studies as for example the Moving Epidemic Method [32]. Secondly, they cannot be considered as monitoring tools (i.e a prospective detection method) but only as retrospective technics allowing the analysis of historical time series. Thirdly, because CPA algorithms are able to identify subtle changes [31], they may be confused by some particular sequences within the baseline, considering them as change points (see 4.1.2 above). If the first important change they detect in the time series is a decrease they may even consider erroneously this low level of the baseline as the signal to detect. This blind change detection is an inaccuracy that cannot be observed when the series are processed by Experts, because they know they have to identify only changes corresponding to increases of the number of cases. This erroneous CPA behavior could be avoided if it would be possible to add a knowledge defining the state to detect (i.e. number of cases during the epidemic state greater than the baseline state). This work explores the results of each algorithm but it should be interesting, first to confirm our results by evaluating algorithms with real data (probably more complex to analyse), and second, with the goal of improving the result accuracy, to combine the results of two or several algorithms, taking in account their specific skills.

## Conclusions

In conclusion, for the aim of retrospectively identifying the start and end of a disease outbreak, in the absence of human resources available to do this work, we recommend using the Kernel change point model. And in case of experts’ availability, we also suggest to supplement the Human expertise with this kind of technics, especially when the SND is below 0 (in practice when the signal is considered as difficult to identify by the experts).

### Availability of supporting data

Data are simulated from a real outbreaks of Norovirus already published [23].

## Additional file

Additional file 1: Supplementary material. (DOCX 1794 kb)

## Abbreviations

CPA: change point analysis
ITSM: inverse transform sampling method algorithm
KFDR: kernel fisher discriminant ratio
PCC: probability of correct classification
SND: signal noise difference

## References

[1] CDC. Glossary. Available: http://www.cdc.gov/OPHSS/CSELS/DSEPD/SS1978/Glossary.html#E. Accessed 14 June 2015

[2] Buckeridge DL (2007). Outbreak detection through automated surveillance: A review of the determinants of detection. J Biomed Inform.

[3] Lawson AB, Kleinman K. Spatial and Syndromic Surveillance for Public Health. Chichester, UK : John Wiley amp; Sons; 2005. http://onlinelibrary.wiley.com/book/10.1002/0470092505.

[4] Wagner MM, Moore AW, Aryel RM. Handbook of Biosurveillance. Amsterdam ; Boston: Academic Press; 2011. http://www.sciencedirect.com/science/book/9780123693785. https://www.library.yorku.ca/find/Record/2271215.

[5] Andrews JM, Langmuir AD (1963). The philosophy of disease eradication. Am J Public Health Nations Health.

[6] Aylward B, Hennessey KA, Zagaria N, Olivé JM, Cochi S (2000). When is a disease eradicable? 100 years of lessons learned. Am J Public Health.

[7] Dowdle WR (1998). The principles of disease elimination and eradication. Bull World Health Organ.

[8] Heymann DL, Rodier G (1997). Reemerging pathogens and diseases out of control. Lancet.

[9] Soumahoro MK, Boelle PY, Gauzere BA, Atsou K, Pelat C, Lambert B (2011). The Chikungunya epidemic on La Reunion Island in 2005-2006: a cost-of-illness study. PLoS Negl Trop Dis.

[10] Brookmeyer R, Stroup DF (2004). Monitoring the health of populations : statistical principles and methods for public health surveillance.

[11] Farrington CP, Andrews NJ, Beale AD, Catchpole MA (1996). A Statistical Algorithm for the Early Detection of Outbreaks of Infectious Disease. J R Stat Soc A Stat Soc.

[12] Siegrist D, Pavlin J (2004). Bio-ALIRT biosurveillance detection algorithm evaluation. MMWR Morb Mortal Wkly Rep.

[13] Watkins RE, Eagleson S, Hall RG, Dailey L, Plant AJ (2006). Approaches to the evaluation of outbreak detection methods. BMC Public Health.

[14] Buckeridge DL, Burkom H, Campbell M, Hogan WR, Moore AW (2005). Algorithms for rapid outbreak detection: a research synthesis. J Biomed Inform.

[15] Chen J, Gupta AK. Parametric statistical change point analysis. 2nd ed. New York: Birkhäuser; 2011. http://www.springer.com/la/book/9780817648008.

[16] Emad-Eldin AAA, Sana SB (1996). Rank tests for two change points. Comput Stat Data Anal.

[17] Held L, Hofmann M, Hohle M, Schmid V (2006). A two-component model for counts of infectious diseases. Biostatistics.

[18] Fricker RD (2013). Introduction to statistical methods for biosurveillance : with an emphasis on syndromic surveillance.

[19] Unkel S, Farrington CP, Garthwaite PH, Robertson C, Andrews N (2012). Statistical methods for the prospective detection of infectious disease outbreaks: a review. J R Stat Soc A Stat Soc.

[20] Chen J, Gupta AK (2011). Parametric statistical change point analysis, 2nd ed..

[21] R Core Team (2014) (2014). R: A language and environment for statistical computing.

[22] Jackson ML, Baer A, Painter I, Duchin J (2007). A simulation study comparing aberration detection algorithms for syndromic surveillance. BMC Med Inform Decis Mak.

[23] Centers for Disease C, Prevention (2000). Outbreaks of Norwalk-like viral gastroenteritis--Alaska and Wisconsin, 1999. MMWR Morb Mortal Wkly Rep.

[24] Harchaoui Z, Moulines E, Bach FR. Kernel Change-point Analysis. In: Koller D, Schuurmans D, Bengio Y, Bottou L, editors. Advances in Neural Information Processing Systems 21: Curran Associates, Inc.; 2009. p.609-16. http://papers.nips.cc/paper/3556-kernel-change-point-analysis. https://scholar.google.fr/citations?view_op=view_citation&hl=fr&user=yCyRTsAAAAJ&citation_for_view=yCyR-TsAAAAJ:d1gkVwhDpl0C.

[25] Yao Q (1993). Boundary-Crossing Probabilities of Some Random Fields Related to Likelihood Ratio Tests for Epidemic Alternatives. J Appl Probab.

[26] Chib S (1998). Estimation and comparison of multiple change-point models. J Econ.

[27] Park JH (2010). Structural Change in U.S. Presidents' Use of Force. Am J Polit Sci.

[28] Martin AD, Quinn KM, Park JH (2011). MCMCpack: Markov Chain Monte Carlo in R. J Stat Softw.

[29] Debin M, Souty C, Turbelin C, Blanchon T, Boelle PY, Hanslik T (2013). Determination of French influenza outbreaks periods between 1985 and 2011 through a web-based Delphi method. BMC Med Inform Decis Mak.

[30] Kass-Hout TA, Xu Z, McMurray P, Park S, Buckeridge DL, Brownstein JS (2012). Application of change point analysis to daily influenza-like illness emergency department visits. J Am Med Inform Assoc.

[31] Taylor WA. Change-Point Analysis: A Powerful New Tool For Detecting Changes. Available: http://www.variation.com/files/articles/changepoint.pdf. Accessed 20 June 2015

[32] Vega T, Lozano JE, Meerhoff T, Snacken R, Mott J, Ortiz de Lejarazu R (2013). Influenza surveillance in Europe: establishing epidemic thresholds by the moving epidemic method. Influenza Other Respi Viruses.

