# Supplementary material for: Outbreak definition by change point analysis: a tool for public health decision?
Source: BMC Med Inform Decis Mak. 2016 Mar 12;16:33. doi: 10.1186/s12911-016-0271-x (PMC4788889; doi:10.1186/s12911-016-0271-x)
Supplement: Supplementary file 1 — Supplementary material. (DOCX 1794 kb) [file 12911_2016_271_MOESM1_ESM.docx]

**Appendix:**

# Figure S1: Expert time series

S1.a: Baseline level = 3 - Outbreak Cases = 30


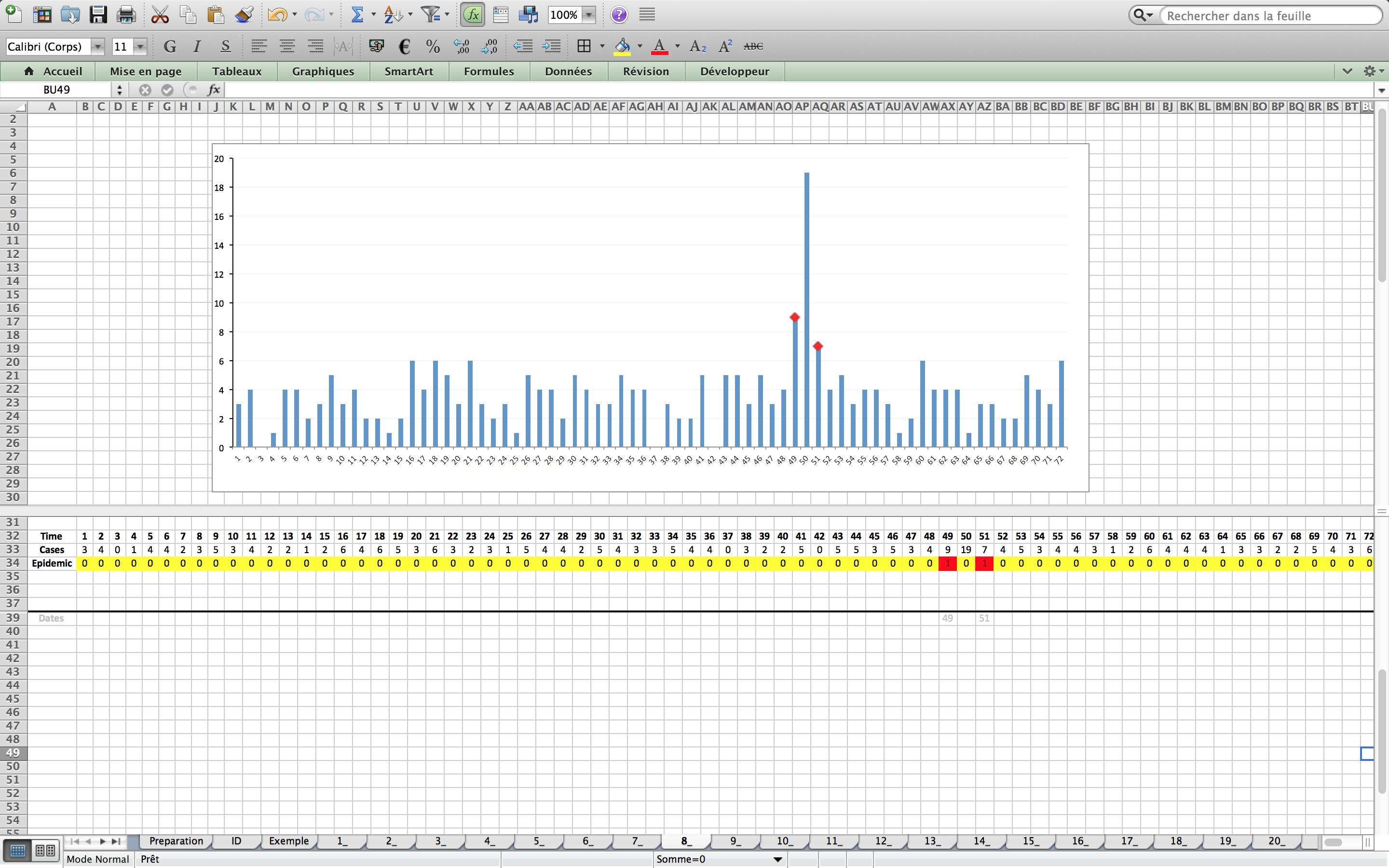


S1.b: Baseline level = 1 - Outbreak Cases = 50


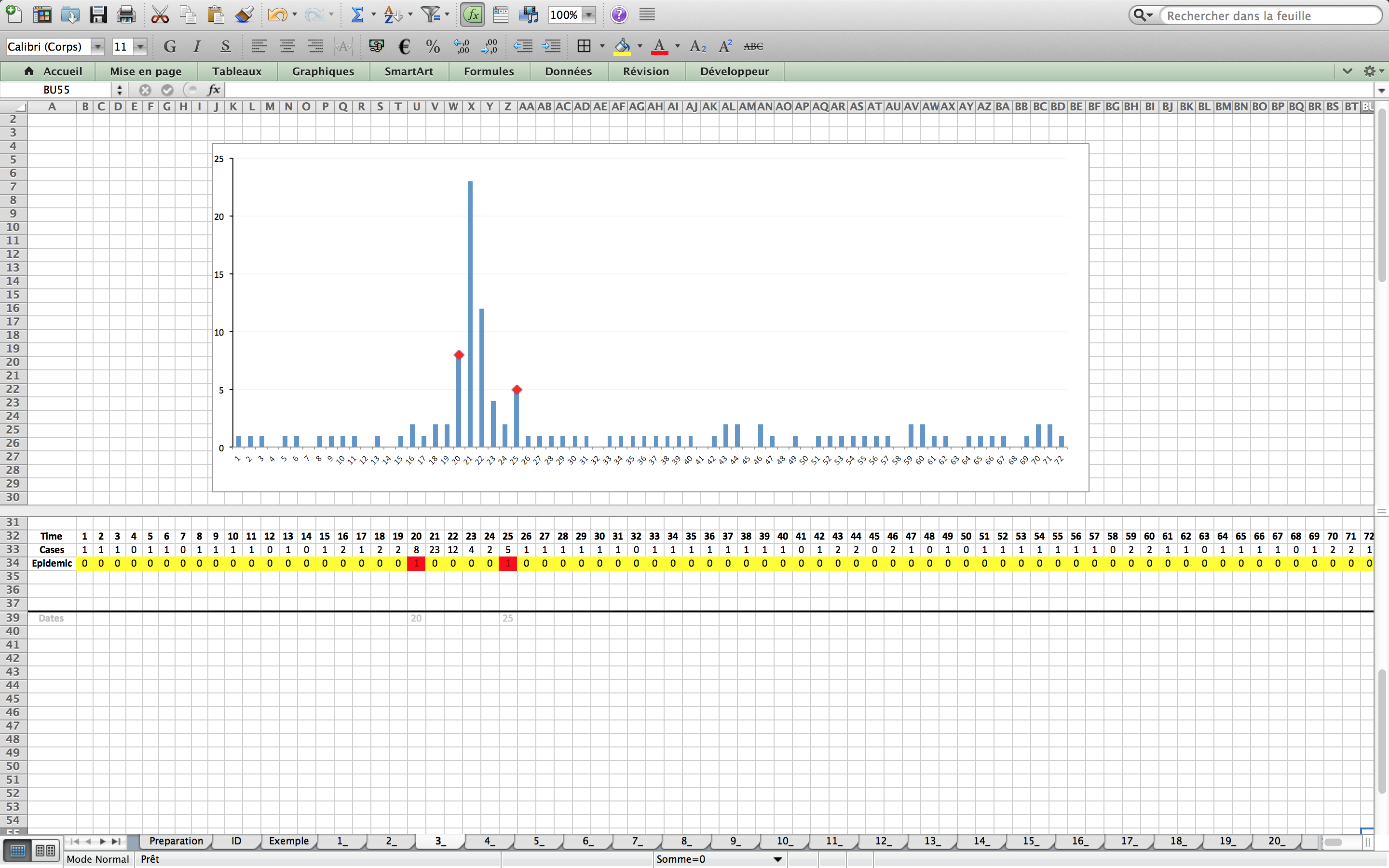


S1.c: Baseline level = 30 - Outbreak Cases = 100


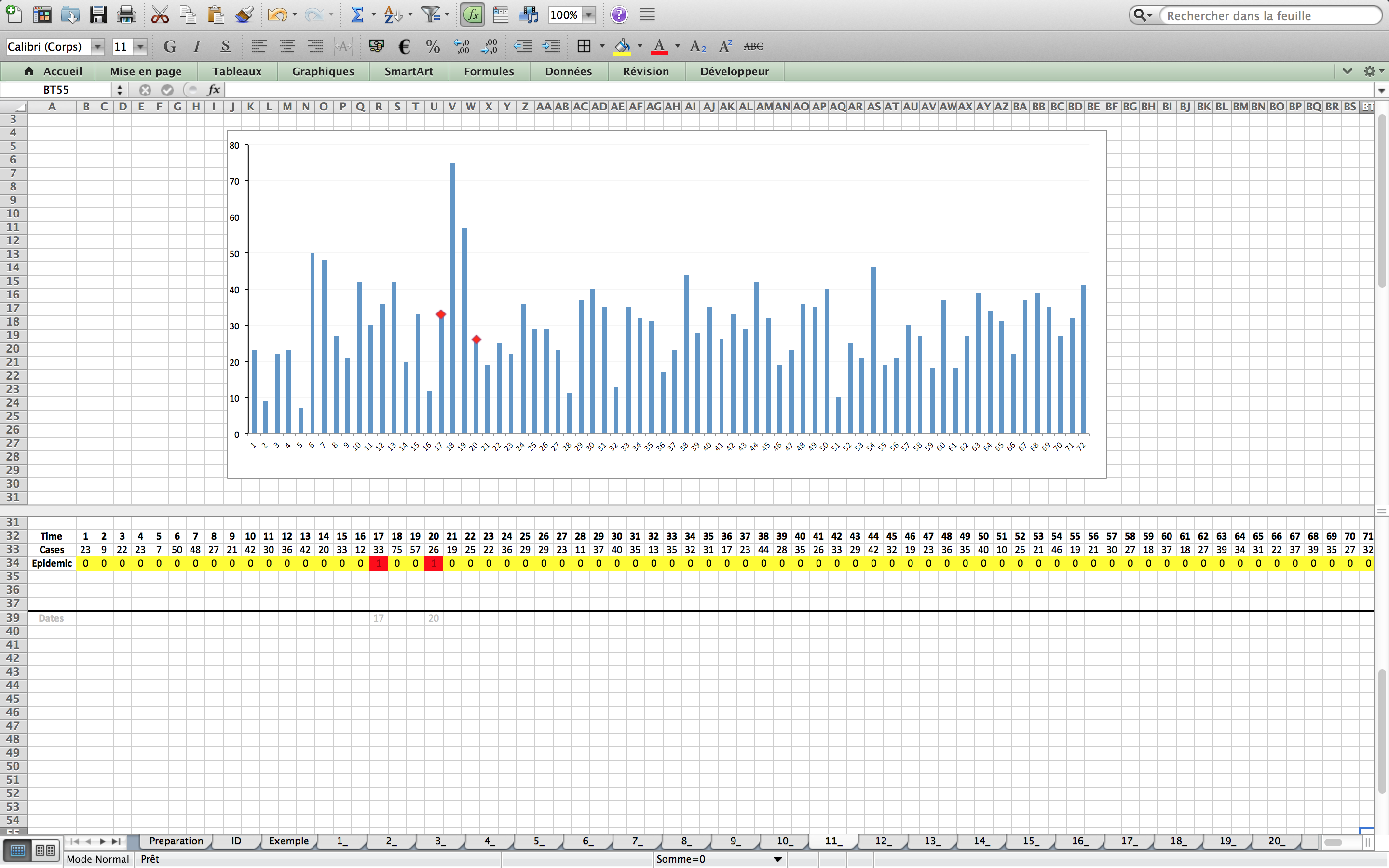


# Figure S2: Algorithms (Maximum Likelihood, Kernel, Kruskall-Wallis, Bayesian, Human) specificity and sensibility evaluation according number of cases in the outbreak (Fig 2a) and level of baseline (Fig 2b)


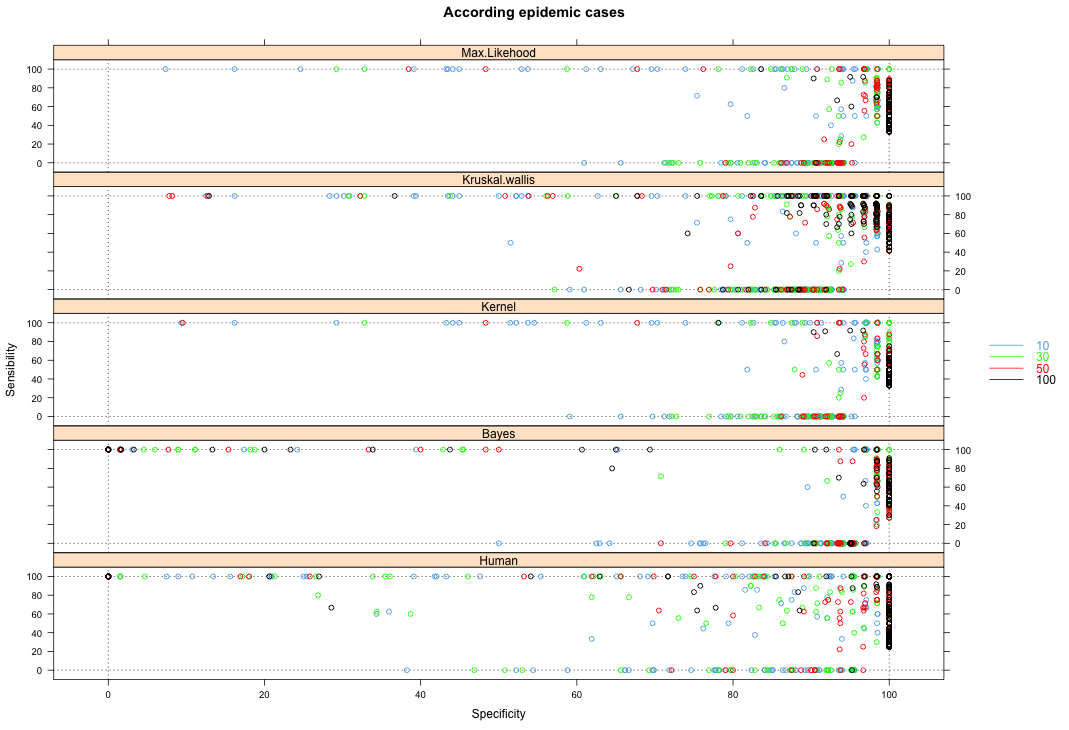


a.


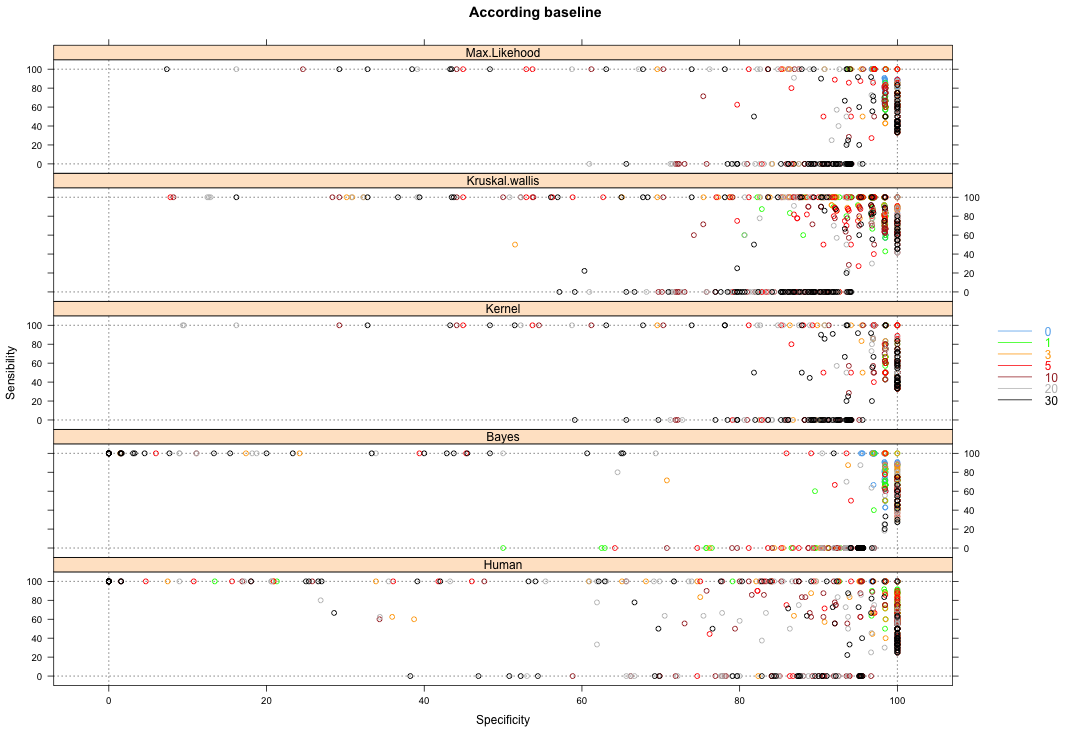


b.

Table S1 – Algorithm bias (mean error) and accuracy (standard deviation) according to the Signal noise difference.

|  | **Max- likelihood** | **K- Wallis** | **kernel** | **Bayes** | **Expert** |
| --- | --- | --- | --- | --- | --- |
| **SND class** |  |  |  |  |  |
| (-350,-300­­] | 24.1 *(20.9)** | 15.3 *(13.1)* | 18.4 *(18.2)* | 37.9 *(23.3)* | 27.7 *(27.1)* |
| (-300,-250­­] | 18.4 *(19.0)* | 17.0 *(19.8)* | 19.3 (20.4) | 36.8 *(24.4)* | 27.1 *(24.7)* |
| (-250,-200­­] | 19.6 (*16.4*) | 21.2 (*17.9*) | 16.8 (17.2) | 42.7 (21.5) | 20.6 (25.4) |
| (-200,-150­­] | 19.5 (*17.5*) | 24.3 (*19.4*) | 15.7 (16.1) | 43.9 (22.6) | 21.7 (20.8) |
| (-150,-100­­] | 22.5 (*19.6*) | 22.8 (*17.9*) | 20.7 (18.0) | 37.9 (23.6) | 29.0 (24.2) |
| (-100,-50­­] | 19.0 (*18.6*) | 23.7 (*20.8*) | 16.5 *(17.8)* | 38.0 (23.6) | 23.8 (22.3) |
| (-50,0­­] | 14.5 *(17.7)* | 16.6 *(18.6)* | 11.1 *(14.7)* | 36.4 *(25.1)* | 19.8 *(24.8)* |
| (0,50­­] | 4.0 *(4.9)* | 5.4 *(8.9)* | 4.5 *(3.5)* | 9.0 *(16.3)* | 4.0 *(8.1)* |
| (50,100­­] | 4.8 *(1.5)* | 3.3 *(3.3)* | 5.7 *(1.4)* | 4.5 *(1.6)* | 3.8 *(3.1)* |
| **Total** | 5.6 *(7.9)* | 6.5 *(8.6)* | 4.9 *(7.0)* | 11.9 *(12.6)* | 7.5 *(12.2)* |

* mean of total error (*standard deviation*) . δ_1_ = Beginning date difference */* δ_2_*=* end date difference

# Parameters estimation

## Maximum likelihood CPA model

Let {x_1_, x_2_,..., x_n_} a time series of *independent* random variables following a Poisson law P.

Under the alternative hypothesis H_1_, we can define 3 segments:

S_1_ = {x_1_, x_2_,..., x_τ1_} ~ P (λ_1_) : pre-epidemic

S_2_ = {x_τ1+1_, x_τ1+2_,..., x_τ2_} ~ P (λ_2_) : epidemic

S_3_ = {x_τ2+1_, x_τ2+2_,..., x_n_} ~ P (λ_3_) : post-epidemic

driving to the estimation of 5 parameters: τ_1_, τ_2_, λ_1_, λ_2_, λ_3_.

As {x_i_}_i=1,2,...,n_ are coming from a Poisson law :

$$P\left( x,\lambda\right)= \frac{exp\left( -\lambda\right).\lambda^{x}}{x!}$$

The time series likelihood, with 2 change-points τ_1_ and τ_2_, can be written:

*L(x_1_, x_2_,…, x_n_⏐ λ_1_, λ_2_, λ_3_, τ_1_, τ_2_)* $=\prod_{i=1}^{\tau_{1}} P\left( x_{i},\lambda_{1} \right).\prod_{i=\tau_{1}+1}^{\tau_{2}} P\left( x_{i},\lambda_{2} \right). \prod_{i=\tau_{2}+1}^{n} P\left( x_{i},\lambda_{3} \right)$

and the Log-likelihood H_1_ can be written:

$$logL= \left( log\lambda_{1}\sum_{i=1}^{\tau_{1}} x_{i} \right)-\tau_{1}\lambda_{1}+ \left( log\lambda_{2}\sum_{i=\tau_{1}+1}^{\tau_{2}} x_{i} \right)-{(\tau_{2}-\tau}_{1})\lambda_{2}+ \left( log\lambda_{3}\sum_{i=\tau_{2}+1}^{n} x_{i} \right)-({n-\tau}_{2})\lambda_{3}-\sum_{i=1}^{n} {log(x}_{i}!)$$

by deduction, in a Poisson law, λ_1_, λ_2_, λ_3_ the values maximizing the Log-likelihood are:

$$\hat{\lambda_{1}}=\frac{1}{\tau_{1}}\sum_{i=1}^{\tau_{1}} x_{i} , \hat{\lambda_{2}}=\frac{1}{\tau_{2}-\tau_{1}}\sum_{i=\tau_{1}+1}^{\tau_{2}} x_{i} , \hat{\lambda_{3}} =\frac{1}{n-\tau_{2}}\sum_{i=\tau_{2}+1}^{n} x_{i}$$

and Log-likelihood can be written with parameter depending only of τ1, τ2 and data observed {x_1_, x_2_,..., x_n_}.

H_0_ (hypothesis in absence of epidemic, with no change point as defined in paragraph 2.2.1) is rejected if Log-likelihood H_1_> Log-likelihood H_0._

We can then identify the couple of point (τ1, τ2) maximizing the Log-likelihood H_1_:

$$\hat{(\tau_{1},\tau_{2})}= {argmax}_{\left( \tau_{1},\tau_{2} \right)\epsilon\left\{ 1,\ldots,n \right\}*\left\{ 1,\ldots,n \right\}}(logL)$$

defining the change point positions τ1 and τ2

## Kernel CPA model

The KFDR between S_1_ and S_2_ is:

$$KFDR\left( S_{1},S_{2} \right)=\frac{mean\left( S_{1} \right)-mean\left( S_{2} \right)}{\frac{(j-i)}{j}Var\left( S_{1} \right)-\frac{i}{j}Var\left( S_{2} \right)}$$

In our case, the start and end dates are identified when i and j are chosen to maximize the heterogeneity between the 3 segments in the following value:

$$V\left( i,j \right)=\frac{i(j-i)}{j}KFDR\left( S_{1},S_{2} \right)+\frac{(j-i)(n-j)}{n-i} KFDR\left( S_{2},S_{3} \right)$$

By analogy with the other CPA models, the couple of point τ_1_, τ_2_ can be obtained by:

$$\hat{(\tau_{1},\tau_{2})}= {argmax}_{\left( \tau_{1},\tau_{2} \right)\epsilon\left\{ 1,\ldots,n \right\}*\left\{ 1,\ldots,n \right\}, \tau_{1}<\tau_{2}}(KFDR\left( \tau_{1},\tau_{2} \right))$$

## Kruskal-Wallis CPA model

If R_i_ is the rank of the observation x_i_ in {x_1_, x_2_,..., x_n_}, the rank sum associated with each segment is:

$$R^{1}=\sum_{1\leq i\leq\tau_{1}} R_{i} R^{2}=\sum_{\tau_{1}+1\leq i\leq\tau_{2}} R_{i} R^{3}=\sum_{\tau_{2}+1\leq i\leq n} R_{i}$$

and the mean :

$$\bar{R^{1}}=\frac{1}{\tau_{1}}\sum_{1\leq i\leq\tau_{1}} R_{i} \bar{R^{2}}=\frac{1}{\tau_{2}-\tau_{1}}\sum_{\tau_{1}+1\leq i\leq\tau_{2}} R_{i} \bar{R^{3}}=\frac{1}{n-\tau_{2}}\sum_{\tau_{2}+1\leq i\leq n} R_{i}$$

The Kruskal-Wallis statistic KW(τ_1_, τ_2_) takes in account the difference between the mean of ranks for each segment and the global mean of all ranks, equal to (n + 1)/2 :

$$KW\left( \tau_{1},\tau_{2} \right)= \frac{12}{n(n+1)}\left( \tau_{1}\left( \bar{R^{1}}-\frac{n+1}{2} \right)^{2}+{(\tau_{2}-\tau}_{1})\left( \bar{R^{2}}-\frac{n+1}{2} \right)^{2}+{(n-\tau}_{2})\left( \bar{R^{3}}-\frac{n+1}{2} \right)^{2} \right)$$

In case of ex-aequos, to each observation belonging to an ex-aequo group we attribute the mean rank of this group, and we calculate the following sum:

$$T= \sum_{j=1}^{h} (t_{j}^{3}-t_{j})$$

Where *t_j_* is the number of element in the j^th^  group and h the number of ex-aequo groups. The new Kruskal-Wallis* statistic is then:

$${KW}^{*}\left( \tau_{1},\tau_{2} \right)= \frac{KW\left( \tau_{1},\tau_{2} \right)}{1-\frac{T}{n^{3}-n}}$$

H_0_ is rejected if the p-value associated with the KW* statistic is <0.05, and the couple of point τ_1_, τ_2_ can be obtained by maximizing the KW* statistic:

$\hat{(\tau_{1},\tau_{2})}= {argmax}_{\left( \tau_{1},\tau_{2} \right)\epsilon\left\{ 1,\ldots,n \right\}*\left\{ 1,\ldots,n \right\}, \tau_{1}<\tau_{2}}({KW}^{*}\left( \tau_{1},\tau_{2} \right))$

## Bayesian model

The challenges of this method reside in the determination of the change points (τ_1_, τ_2_) by estimating the transition probability band matrix as proposed by Chib [1] where:

- p_ij_ = Pr (S_t_ =j|S_t-1_=i) is the change probability for regime j at time t, knowing that at time t-1 the system was at regime i.
- p_ii_ = Pr (S_t_ =i|S_t-1_=i) is the probability to stay at regime i.

with the sum of each line of the matrix equal to 1.

As we want to detect the beginning and the end of the outbreak, we set the number of dates to find at 2 (τ_1_,τ_2_) and the number of hidden states (or regimes) at 3 with $\lambda_{t}$ = {θ_1_,θ_2_,θ_3_} and S_t_ = k (with k =1,2,3), representing the pre-epidemic, epidemic and post-epidemic regimes.

$$P= \left( \begin{matrix} p_{11} & p_{12} & 0 \\ 0 & p_{22} & p_{23} \\ 0 & 0 & 1 \end{matrix} \right)$$

The time series is drawn from the law *f*(X_t_|X_t-1_, θ_k_ ). The count *x_t_* for day *t* is modeled via a hierarchical Poisson model.

$f\left( x_{t} | \lambda_{t} \right)=\frac{\exp\left( -\lambda_{t} \right).{\lambda_{t}}^{x_{t}}}{x_{t}!}$ with t ≤ n

Using conjugate prior distributions, as proposed by Chib [1], Park [2] and Martin [3], we can write a Bayesian Poisson change-point model without covariates as follows, assuming Beta priors for transition probabilities and Gamma priors for λ_i_:

X_t_ ~ Poisson (λ_i_), i = 1,..., k where k is the number of states.

λ_i_ ~ Gamma (c_0_, d_0_)

p_ii_ ~ Beta(a,b), i = 1,...,k.

This model is subject to two breaks with λ_1_, λ_2_, λ_3_~ *Gamma* (c_0_, d_0_). Where c_0_ is the mean and d_0_ = 1.

------------------------------------------------

1. Chib S. **Estimation and comparison of multiple change-point models**. *Journal of Econometrics* 1998, **86(2)**:221-241.

2. Park JH. **Structural Change in U.S. Presidents' Use of Force**. *American Journal of Political Science* 2010, **54(3)**:766-782.

3. Martin AD, Quinn KM, Park JH. **MCMCpack: Markov Chain Monte Carlo in R**. *Journal of Statistical Software* 2011, **42(9)**:1-21.
